# Ahd Hamidi: improving access to vaccine production capacity

**DOI:** 10.2471/BLT.19.031219

**Published:** 2019-12-01

## Abstract

Ahd Hamidi talks to Gary Humphreys about technology transfer and the implications of new vaccine production methods for local production.

GH: *You describe yourself as a bioprocess technologist. Can you explain what that is?*

AH: Someone who works on the design and development of equipment and processes used to produce different products from biological materials. In my case, vaccines.

GH: *How did you get into that field?*

AH: I studied chemical engineering at the Technical University in Delft, and at the time the degree included a life sciences component. That’s where I first started taking an interest in the immune system and antigen response. I did a master’s degree in industrial microbiology, focusing on antigen production, which is an upstream part of the development process. I then did a second master's, known as a professional doctorate in engineering, focusing on antigen purification, which is a part of the downstream process. So, everything came together in my studies, leading eventually to my focus on vaccines.

GH: *How did you transition from the laboratory to the broader global health space and in particular to working on the issue of access to vaccines?*

AH: After completing my studies, I worked for a short time as a scientist at TUDelft, but then I joined the Rijksinstituut voor Volksgezondheid en Milieu (RIVM) in 1998, which was a major technology transfer hub. At RIVM, I worked on capacity building and technology transfer to developing-country manufacturers. I developed training modules and coordinated some of the trainings of the Global Training Network, which was financed through the World Health Organization. The international department activities were later transferred to the Netherlands Vaccine Institute (NVI) and to the newly-created Institute for Translational Vaccinology (Intravacc), which came out of the merger of the vaccinology unit of RIVM and NVI.

GH: *So, at that point you had left the laboratory?*

AH: No, I continued to work on vaccines as a bioprocess technologist and project manager, developing processes and managing projects solely meant for technology transfer. That helped me later in my discussions with stakeholders as they moved towards vaccine manufacture.

GH: *What were the projects you worked on at RIVM?*

AH: One project was to train manufacturers in the basics of manufacturing and quality control of vaccines, such as DTP (diphtheria, pertussis and tetanus). India was one of the countries that sent people to us. They have since become one of the biggest vaccine manufacturers in the world, having companies such as the Serum Institute of India in Pune. Chinese manufacturers were also supported by RIVM from a very early stage, for example with the technology required for OPV (oral polio vaccine) production. We also trained people in the manufacture of conjugate vaccine, starting with *Haemophilus influenzae* type b (Hib), the first conjugate vaccine used in humans. The vaccine first became available in 1987, but many governments couldn’t introduce it because of the cost. At RIVM we developed a product that had exactly the same quality profile as the vaccines being produced by big pharma, but which could be produced at approximately US$ 1.0 a dose, compared to approximately US$ 3.5 a dose, which was the United Nations Children's Fund (UNICEF) price at that time. We then transferred the production technology to China, India and Indonesia, and they started producing conjugate vaccines that were much cheaper than anything previously available.

“[Emerging market manufacturers] supply about half of UNICEF's vaccine procurement in terms of volume of doses.”

GH: *That must have been very gratifying.*

AH: I’m very proud of the part we played in facilitating the global entry of emerging market manufacturers. They have gone on to make a huge difference to global vaccine supply, and now supply about half of UNICEF's vaccine procurement in terms of volume of doses.

GH: *You left RIVM in 2006 and worked abroad as a consultant. What inspired you to make that big change?*

AH: A desire to see the world was part of it. I grew up in Morocco and didn’t move to the Netherlands until I was 17. So, I had a powerful sense of there being a big world outside my adopted country. Also, I really enjoy communicating and connecting with different people and cultures. But the move I made in 2006 wasn’t just about wanting to see the world. I also wanted to get outside the public sector, and, from a professional point of view, to put myself on the other side of the table – to be a part of a developing country manufacturer’s team.

GH: *Where did you go?*

AH: Everywhere people make vaccines! Or almost everywhere. Of course, in some countries there were a lot of manufacturers, such as India and China, so I spent more time there.

GH: *Do countries differ in terms of the technology transfer challenges they face?*

AH: Everywhere is different, but there were certainly recurring themes in terms of challenges: lack of research and development infrastructure, lack of production capacity and lack of human resources. But I encountered other things too, including things that had nothing to do with the nuts and bolts of technology transfer, but were important nonetheless.

GH: *Can you give examples?*

AH: For example, I learned just how important trust is. There has to be trust between the transferrer and the transferee. And that takes time to establish. You cannot simply come in and say, ‘tomorrow we have to have this’. That doesn’t work. And the other big issue is hierarchy; how power is distributed. My experience is that sometimes the quietest person in the room is the most influential one and is the one who will decide what is going to happen. So you have to be very observant, but you also have to be able to put yourself in the other person’s place to try and see things as they see things.

GH: *Did you have any opportunities to do that?*

AH: Yes. I lived in India for two years and worked for an Indian company. Subsquently, I took on several different assignments, serving one or two months in each. I think that in three years I learned much more about certain aspects of technology transfer, for example in relationship management, than I learned in all the other years of my career. I mean in terms of understanding how things really work and what might be the blocking points in any given project. It’s all very different when you see it from the point of view of the country that is receiving the technology. My experience is that if you knock on the right door you can almost always address any technical or financial aspects; the real obstacles are at the level of management, communication and human relations.

“The real obstacles [to technology transfer] are at the level of management, communication and human relations.”

GH: *You returned to Intravacc, in 2009, but then left again in 2017 to join Batavia, a private company. That must have been a big change after so long in the public sector.*

AH: It certainly was. But in many ways I am continuing my work in global health. Indeed, I joined Batavia because of the work they are doing in that area, notably around vaccine process intensification.

GH: *What does vaccine process intensification mean?*

AH: In the simplest terms, it means organizing the production process in the most efficient way, by simplifying, streamlining and where possible, integrating process steps. The main aims of process intensification are to reduce the amount of equipment used and the space taken up by the production facility, while also reducing processing complexity and therefore costs and risks.

GH: *Can you describe a current project?*

AH: We are currently working on a platform that involves a new kind of bioreactor. That’s the container used to make the desired biological product, in this case, a vaccine. The bioreactor was developed by a company called Univercells which is our consortium partner in a project that was funded by the Bill & Melinda Gates Foundation (BMGF) in 2016. The main advantages of the bioreactor is a reduced footprint and ease of operation, both of which have implications for cost.

GH: *What kind of cost reduction can be achieved?*

AH: Savings will vary from vaccine to vaccine. But to give you an idea, Sabin inactivated polio vaccine (sIPV) was used as the first target vaccine under the BMGF grant.

GH: *You mean that was the vaccine you produced using the intensified process?*

AH: That’s right. So, according to our modelling of the process, which was developed at the research and pilot scale, the estimated cost per dose for trivalent sIPV using the intensified manufacturing platform is less than US$ 0.30 per dose. That is a fifth of the current UNICEF price for this vaccine. We estimate that the vaccine could be produced in a micro-facility, costing between US$ 30-40 million and capable of delivering around 50 million trivalent doses per year, compared to around US $100 – 150 million, which would be the usual level of investment required to go into production at that scale. Batavia will be using the platform to manufacture clinical lots of sIPV. It will be supplied to developing country vaccine manufacturers to accelerate clinical testing and licensure. Production is scheduled to start in 2020.

GH: *Are there benefits other than reduced costs?*

AH: Intensified processes in small foot-print facilities also allow for a significant reduction in turnaround time. So, a manufacturer could be more responsive to sudden increases in demand for a given vaccine. Also, the modular nature of the platform allows for adaptation according to need. We are currently working on testing this platform for measles and rubella. Going forward we intend to explore the platform’s capabilities in optimizing the responsiveness to outbreak situations and for producing high-quality biologics.

GH: *How close are you to being able to share this technology with developing countries?*

AH: We have already begun discussions with different manufacturers regarding the terms of agreements and collaborations. We are at the beginning of that process.

